# Euregional Youth Survey Structure and results of the cross border youth surveys

**DOI:** 10.1093/eurpub/ckac129.214

**Published:** 2022-10-25

**Authors:** D Philippsen

**Affiliations:** Department of Public Health Reporting, Gesundheitsamt Kreis Düren, Düren, Germany

## Abstract

The Euregional Youth Study takes place at intervals of currently 4 years since 2001. The Dutch, German and Belgian municipalities of the Euregio Meuse-Rhine (EMR) can participate. Pupils in the 8th and 10th grades are asked about various topics in an online questionnaire. These include: physical and emotional well-being, physical activity, nutrition, media behaviour, drug use and school behaviour. In 2019, 88 schools with more than 13,500 participants took part. With its cross-border approach, the study provides the opportunity to compare the living conditions, behaviour and health situation of pupils in the three countries. Ideally, this would result in common policy and prevention approaches and best practice options. For example, there are differences between the regions of the EMR regarding drug use or overweight, while risky media use is rather universal. It is striking that the Dutch participants almost consistently show the best values. It is also important to stress the importance of insight in policy along the border. Changes in policy actions have a huge effect on border regions. Examples are:

The change in drinking age in the Netherlands: from 16 to 18 resulted in organizing their parties in the neighbouring countries.

The change in cannabis policy in the Netherlands in the 2000s, is clearly reflected in the purchasing behaviour of German young people.

Independent of the cross-border aspects, the Euregional Youth Survey provides a standard data set (also with trends over time) for the adolescents of the participating districts, which the local health offices could not realise on their own and which is not self-evident for German municipalities.On the other hand, it is certainly considered as problematic that there is no binding and uniform participation of the EMR partners in the study. This leads to a partial loss of comparability and significance of the Euregional Youth Study.

